# Pain Assessment: Benefits of Using Pain Scales for Surgical Patients in South Bohemian Hospitals

**DOI:** 10.3390/healthcare9020171

**Published:** 2021-02-05

**Authors:** Vera Olisarova, Valerie Tothova, Martin Cerveny, Vendula Dvorakova, Petr Sadilek

**Affiliations:** 1 Institute of Nursing, Midwifery and Emergency Care, Faculty of Health and Social Sciences of University of South Bohemia, 37001 České Budějovice, Czech Republic; tothova@zsf.jcu.cz (V.T.); cervem13@zsf.jcu.cz (M.C.); 2 Hospital České Budějovice a.s., 37001 České Budějovice, Czech Republic; VenDvorakova@seznam.cz; 3 Medical Information Centre, 11000 Prague, Czech Republic; sadilek@help-lic.cz

**Keywords:** pain, pain management, pain assessment, SF-MPQ-2, surgical patients

## Abstract

Pain is a medical and nursing problem that is common in surgical departments. Inadequate pain management can lead to patient distress, as well as extending the period in which the patient’s quality of life is reduced. The standardized SF-MPQ-2 questionnaire provides nurses with the opportunity to assess pain within a broader context. The aim of this descriptive and exploratory study was to describe the state of pain assessment in surgical patients in the South Bohemian Region and to highlight the benefits of using a standardized tool for proper pain assessment. The research was carried out using a quantitative survey within the South Bohemian Region (Czech Republic). The participants in the study were nurses working in surgical departments in hospitals in the region as well as hospitalized patients. The results show that nurses pay slightly more attention to pain assessments than doctors. We know that, generally, pain decreases with time after surgery. Nonetheless, returning pain, as well as continuous pain, can occur, both of which have an emotional component. The results of this study are directed at nurses and include a call for more effective pain management through improved assessment.

## 1. Introduction

Pain is a phenomenon that mankind has had to endure for an immemorial period of time. Surgery is one field of medicine in which health professionals frequently have to deal with patient pain. In most cases, pain is related to injuries and diagnostic procedures, but it is also often associated with surgical procedures. Most patients hospitalized in surgical wards experience some level of pain [1]. If pain management is inadequate, patient experience can be very poor. This can lead to discomfort, sleep disturbances (especially waking up due to the pain), increased treatment duration, an increased time during which the patient’s quality of life is reduced, and a delay in returning to everyday life [2,3,4].

This study describes the manifestation of pain and its effects on patients (N = 205) hospitalized in surgical departments in the South Bohemian Region (Czech Republic). The most frequently mentioned aspects are physical manifestations, movement restrictions, decreased self-sufficiency, emotional instability, lowered verbal expression, and grimacing due to pain [5]. Pain management still poses a significant challenge for doctors, nurses, and other medical staff.

Although progress in scientific, pharmacological, and technological areas has improved our ability to dampen pain, we cannot say that the problem of pain management has been completely solved [6]. The first step in providing adequate pain control is the correct evaluation of pain. Today, a number of assessment tools are available to assess pain, including visual scales for pain assessment, the critical care observation tool (CPOT), the face, leg, activity, cry, and consolability scale (FLACC), and the behavioral pain scale (BPS). In the South Bohemian Region (Czech Republic), based on data obtained from patients hospitalized in surgical departments (N = 205) and nurses working in this type of ward (N = 253), the visual analogue scale, verbal pain assessment, and pain assessment records are the most commonly used assessment tools [5]. These assessment tools are single-dimensional, i.e., these scales measure only pain intensity. They provide information about the pain intensity score over time. However, in Czech Republic surgery departments, there are patients with a wide spectrum of diagnoses (i.e., injuries, patients with planned surgery, those with a cancer diagnosis, etc.). This single-dimensional measurement method could be unsatisfactory because information about emotional aspect could be missed. Pain is a phenomenon with a holistic impact, and for effective pain management, a holistic evaluation is necessary.

The essential requirements for any evaluation scale include validity, availability, sensitiveness, the suitability for routine use and the ability of health professionals to work with these tools [6,7]. Furthermore, it is necessary to consider factors such as patient age, patient vision, and the patient’s ability to understand the assessment tool [8]. A study published by Mustajoki, Forsén, and Kauppila [9] showed that language barriers relative to the language of the standardized instrument (in this case, SF-MPQ) could lead to inadequate pain management, especially in the affective component. For proper pain management, it is essential that nurses administer assessment tools according to established standards [7].

One of the most widely used pain assessment tools worldwide is the McGill Pain Questionnaire, which is available in both long-form and short-form formats. Since the long-form questionnaire contains a total of 78 descriptors, it can be very time consuming, requiring at least 20 min. In contrast, the original short version of the questionnaire only requires about 5 min [10]. The abbreviated version was modified to account for the needs of various target groups as well as for use in different countries (i.e., language variations). As a result, there are some differences between the different versions of the questionnaire, especially regarding the translation of specific words. Need for improved pain management was the main reason for the modification of the abbreviated version. It showed very good levels of acceptability and comprehension in initial patient testing [11,12,13,14,15,16].

## 2. Materials and Methods

### 2.1. Study Design

This descriptive and exploratory study focused on describing the state of pain assessment in surgical patients in the South Bohemian Region and examined the potential for using the McGill Pain Questionnaire (short-form) (SF-MPQ) to evaluate pain in a group of surgical patients. Quantitative methods in the form of questionnaire surveys were used to achieve this objective.

### 2.2. Study Participants

Study participants were chosen non-randomly based on predetermined selection criteria. These criteria were in line with the objectives of the study, i.e., focused on patients hospitalized in surgical departments and nurses working in surgical departments of medical institutions in the South Bohemian Region. The selection criteria also had a demographic element, i.e., the South Bohemian Region of the Czech Republic, and an age element, i.e., patients had to be aged ≥15 years and hospitalized in a surgical department. The criterion for nurses was that they were working on a surgical ward. The sample size for the study was determined by a statistician and was based on the number of medical facilities in the South Bohemian Region and the number of surgical departments in these facilities.

In the South Bohemia region, there are eight medical facilities. The research team found out the number of hospitalized patients in surgical departments per year in the South Bohemia Region from the available statistics data. Additionally, we found the number of nurses in this region, but the number of nurses working in the surgery departments in each of these medical facilities is not available. These details have not been made open access. Therefore, the sample size of nurses working in surgical department was determined by a statistician based on available data. This means that we started from the number of surgery departments and statutory minimum number of staff in health services, which is given by Decree of Ministry of health Czech Republic 99/2012 Sb.

A total of 320 questionnaires were distributed to the nurses working in surgical departments of medical facilities in the South Bohemian Region (Czech Republic); of these, 253 (79.06%) completed questionnaires were returned. Additionally, 320 questionnaires were distributed to patients hospitalized in the surgical departments of medical institutions in the South Bohemian Region; of these, 218 (68.13%) questionnaires were returned. Of the 218 questionnaires returned, 13 questionnaires had to be excluded due to incompleteness. The resulting research group consisted of 253 nurses working in the surgical department of a medical facility in the South Bohemian Region (male = 6 (2.4%) and female = 247 (97.6%)). The resulting set of hospitalized patients included 205 respondents (male = 91 (44.4%) and female = 114 (55.6%)).

### 2.3. Data Collection

The quantitative research survey took place from 1 March 2019 until the end of April 2019 in the surgical departments of medical institutions in the South Bohemian Region (Czech Republic). It was carried out using two sets of questionnaires. The first questionnaire was given to patients hospitalized in surgical departments of medical institutions in the South Bohemian Region and contained a standardized part and a non-standardized part (i.e., questions of our own design). The questionnaire was distributed by members of the research team (nurses). Patients were acquainted with the questionnaire. If they had any questions about the questionnaire, they were able to ask nurses who were members of the research team. The questionnaire was constructed as a self-evaluation questionnaire. In cases where a patient had a problem with reading (deterioration of visual abilities), a member of the research team asked the questions face-to-face.

The second questionnaire was given to nurses working in surgical departments of medical institutions in the South Bohemian Region. The questionnaire was distributed by members of the research team. If the nurses had any questions about the questionnaire, they were able to ask nurses who were members of the research team. This questionnaire only contained our in-house questions.

### 2.4. Instrument Characteristics

#### 2.4.1. Questionnaire Given to Patients Hospitalized in Surgical Departments of Medical Institutions in the South Bohemian Region

This questionnaire contained 43 numbered questions. It was made up of a standardized part (questionnaire SF-MPQ-2) and a non-standardized part (i.e., questions of our own design). The non-standardized section contained closed and semi-closed (with the ability to write in custom answers) filtering, and scale-based questions.

The non-standardized questions (i.e., questions of our own design) were created based on the relevant literature and the experience of the research team. The first three questions were to do with categorization. The next 13 questions were focused on ascertaining felt pain. We used closed, semi-closed, and scale-based questions (in this case numeric pain rating scale: 0–10 integers). We used figures for pain drawing too. The use of these questions gave us the opportunity to describe felt pain, the difference between pain at the time before hospitalization and during the period of hospitalization, the difference between pain before the operation and after the operation, the length of time that the patient felt pain at this intensity, and the impacts of pain. The remaining 27 questions were focused on evaluating pain based on the viewpoint of patients. These questions gave us the opportunity to determine who evaluates patients’ pain, determine the scales used for pain assessment, and determine the levels of patients’ satisfaction with pain assessment in the surgery department and cooperation with physician and nurses. Subsequently, the intelligibility of the questions was verified within the framework of a pilot study carried out using 5 patients hospitalized in the surgical department of a medical facility in the South Bohemian Region. Based on their replies, the questionnaire was modified in cooperation with a statistician. The questionnaire was finalized after the consideration of all recommendations and comments.

The standardized part was made up of the short-form of the McGill Pain Questionnaire (SF-MPQ-2). This questionnaire was translated into Czech and pre-standardized in 1990 by Šolcová et al. [14]. In 2009, it was further modified to clarify and delineate the pain experienced. The main modification was the addition of descriptors. In the new Czech version, there are 22 descriptions [15]. Consent to use the Czech version of the questionnaire (including the procedure for its evaluation) was obtained from the Mapi Research Trust. The standardized SF-MPQ-2 questionnaire assesses not only pain intensity but also the quality or nature of a patient’s pain. It contains a visual analogue as well as a verbal assessment of pain intensity. Its abbreviated version contains pain descriptors divided into fifteen categories. The first eleven descriptors in the questionnaire are associated with the sensory subscale; the remainder are associated with the affective subscale. Both parts of the questionnaire are scored, and when combined, they yield a Pain Rating Index [10,14].

#### 2.4.2. Questionnaire Addressed to Nurses Working in Surgical Departments of Medical Facilities in the South Bohemian Region

This questionnaire contained only in-house questions of our own design. These questions were created based on theoretical knowledge, studied literature, and the experience of the research team. The intelligibility of the questionnaire addressed to nurses was verified in a pilot study carried out with 4 nurses working in surgical departments in the South Bohemian Region. According to their comments and suggestions, the questionnaire was subsequently modified and finalized in cooperation with a statistician. The resulting questionnaire contained 31 questions. The questionnaire contained closed, semi-closed (with the possibility of entering custom answers), filtering, and scale-based questions. The first four questions involved categorization. The next 27 were focused on pain evaluation from the viewpoint of nurses working in the surgery department. These questions gave us the opportunity to determine who evaluates patients’ pain, how patient pain is evaluated, what scales are used for pain assessment, if nurses are content with the scales being used and what they would like to add into these scales, how objective symptoms of pain are identified in patients, and the level of satisfaction with cooperation between patients and physicians on pain management.

### 2.5. Ethical Considerations

This study did not include any ethically contentious issues. The implementation of the study respected Regulation 2016/679 of the European Parliament and the Council of the EU, formed on 27 April 2016 for the protection of individuals during the processing of personal data and the free movement of such data, repealing Directive 95/46ES (General Data Protection Regulation). All activities related to the inclusion of human subjects in research were carried out in accordance with the 1975 Helsinki Declaration and its latest revision in 2013 and were in accordance with national ethics standards and regulations.

Respondents were familiarized with the questionnaires and goals of the study, and the study was conducted anonymously. Participation was voluntary. Respondents agreed to participate by completing the questionnaire. The introduction section of the questionnaires explained the purpose of the study and provided information regarding the potential advantages and disadvantages associated with completing the questionnaire. Both questionnaires were examined and approved by the Ethics Committee of the University of South Bohemia. For the implementation of the study, approval and consent from the relevant surgical departments and medical institutions were also obtained, as was the consent of the nurse supervisors at these medical institutions.

### 2.6. Data Analysis

After visual inspection, data obtained (from the questionnaire addressed for patients and questionnaire addressed for nurses) were evaluated using SASD version 1.4.10 (SPIROX, s.r.o., Prague, Czech Republic) and SPSS version 24 (ACREA CR, s.r.o., Prague, Czech Republic). Frequency tables were created for each indicator, as well as absolute and relative frequencies, modes, medians, averages, variances, and standard deviations (first degree data sorting).

For each variable, a variance estimate, standard deviation estimate, range, interval mean estimate of 0.05, and an interval variance estimate of 0.05 were also calculated. Pivot tables with absolute and relative frequencies and a sign scheme were constructed as part of second degree data sorting. As part of the analysis of relationships, tests were applied according to the nature of the distribution of variables and the frequency of observations.

Data analysis of the standardized questionnaire SF-MPQ-2 was carried out using procedure established for this standardized tool in SPSS. To evaluate the overall SF-MPQ-2 score and its individual domains in terms of the strength of relationships with specific variables, it was necessary to first test the distribution of variables using the Kolmogorov–Smirnov and Shapiro–Wilk normality tests. Based on the results that showed that the variables did not have a normal distribution, non-parametric tests (Kruskal–Wallis test, Spearman’s Rho non-parametric test) were used to test statistically significant links.

## 3. Results

A total of 253 nurses working in the surgical departments of medical institutions in the South Bohemian Region and 205 hospitalized patients participated in the survey. Among nurses, significantly more were women (247 (97.6%)), and age categories were similarly represented. Regarding the highest education level attained and the length of health care experience, the distributions were uneven.

As with nurses, there were more female patient respondents (114 (55.6%)). Most reported that they had only been hospitalized once over the past year, and most were on post-surgical days 1 or 2. There were 43 pre-surgical patients (Table 1).

For the present state of pain assessment, it was important to define who was responsible for evaluating pain in the surgical department. Therefore, we asked nurses who evaluated pain in their department. The analysis of the nurses’ responses (N = 253) indicated that the assessment of pain in surgical departments is carried out by nurses (253). At the same time, 77.9% of nurses said that doctors carried out the assessment of pain in their surgical department (197). However, in the analysis of responses received from patients, 185 patients (90%) indicated that they had been assessed by a physician, 195 (95%) had been assessed by a nurse, and 17% (34) of patients also mentioned a physiotherapist.

Pain assessment is one of the nurse’s competencies, as is the choice of an adequate assessment tool. The visual analog scale was shown to be one of the most widely used scales for pain assessment. One hundred and twenty-nine nurses stated that they use this scale for the pain evaluation of surgical patients (N = 253). In statistically testing of the relationship between the education of nurses and their evaluation of the adequacy of a visual analog scale. It was found that the nurses with the lowest level of education (secondary school) were significantly more likely to state that they did not know whether the evaluation of pain using a visual analog scale was sufficient (N = 226; X^2^: 40,727; df: 15; *p* < 0.001). Nurses with higher levels of education, namely a university bachelor’s degree, evaluated the visual analog scale as being rather sufficient or moderately sufficient significantly more often. Therefore, we also evaluated the relationship between the education of nurses and their requirements for the assessment of pain supplementation. A statistically significant association was identified (N = 253; X^2^: 38,794; df: 18; *p* < 0.01). Nurses with the lowest level of education (secondary school) stated that it is not necessary to supplement anything in the assessment of pain significantly more often. Nurses with a university (bachelor’s) degree recommended the use of a more accurate verbal description significantly more often.

Patients were also asked about the adjectives that they would use to characterize their pain. To express this characteristic, they were offered a list of five pain-characterizing adjectives with the option of writing in an adjective of their own choice (sharp—shooting—convulsive—persistent—blunt—tiring) with the option of writing in an adjective of their own choice. Patients could choose one or more options. The most frequency options were “sharp” (101), “tiring” (46) and “persistent, blunt” (41).

To express their current state of pain, respondents ranked their pain using a numerical scale of 0 (no pain) to 10 (the greatest pain they had ever experienced). The numerical scale was evaluated as follows: numbers 0–3 mean no current pain, weak pain; numbers 4–6 mean moderate pain and numbers 7–10 mean severe pain, highest possible level of pain. We can state that “no current pain or weak pain” was felt by 51 patients (25%), “moderate pain” was felt by 128 patients (64.2%) and “severe pain, highest possible level of pain” was felt by 26 patients (12.6%).

To determine the extent of relationship between current state of pain and the SF-MPQ-2 assessment, the Spearman’s Rho non-parametric test was used. Statistically significant associations were found between intermittent pain and the overall SF-MPQ-2 score. When describing current pain, patients (N = 205) assessed it mainly as intermittent pain, which was reflected in the total pain SF-MPQ-2 score (Table 2).

Pain is a phenomenon with both psychological and physical components. As part of second level sorting, relationships between individual variables were monitored, namely, between duration and pain intensity and between pain and its impact on a patient’s mental and physical conditions. Patients were offered a five-point scale to assessment the impact: maximum—high—medium—low—none (Table 3). In the analysis of responses received from patients (N = 205), 76 patients (37.1%) stated that pain had “none” impact to their mental state. A total 41 patients (20%) stated “low” impact to their mental state, 37 patients (18%) stated “medium” impact to their mental state. Only 39 patients (19%) stated “high” impact of pain and 12 patients (5.9%) stated “maximum” impact of pain to their mental state. In the analysis of responses received about pain effect on patients’ physical condition, 39 patients (19%) stated that pain had “none” impact to their physical condition. A total 62 patients (30.2%) stated “low” impact, and 48 patients (23.4%) stated “medium” impact to their physical condition. Only 36 patients (17.6%) stated “high” impact of pain and 20 patients (9.8%) stated “maximum” impact of pain to their physical condition.

In the case of the subjective perceived impact of pain (physical and mental impact), patients were asked to subjectively describe their symptoms. Patients were offered answers with the possibility of adding others. For mental impact, the options were aggressiveness, emotional lability, verbal expressions, apathy, I cannot judge, and others (with the possibility of adding other symptoms). For physical impact, the options were limited movement, limited self-sufficiency, facial expressions (or grimacing), I cannot judge, and others (with the possibility of adding other symptoms). Patients could choose one answer or more answers. Other symptoms, which were stated, were then categorized based on the context.

The most common manifestations of the current pain state were emotional lability (59, 33.5%), verbal expression (48, 27.3%), apathy (32, 18.2%), and aggression (12, 6.8%). In terms of maintaining self-sufficiency, patients reported that their current pain state caused a decrease in mobility (140, 48.4%) and limited their degree of self-sufficiency (97, 33.6%).

The relationship between the patient’s mental and physical conditions and their pain was also evaluated using the standardized SF-MPQ-2 (Table 4) questionnaire. The association between the mental state and SF-MPQ-2 results was tested using the Kruskal–Wallis test. Statistically significant differences relative to the influence of the mental state were found in the emotional characteristics of pain and continuous pain as well as in the overall scores; the same was true for continuous moderate pain. It should be noted that the psychological state was found to be negatively affected by continuous pain and the emotional characteristics of pain, causing overall pain (according to the SF-MPQ-2) to have a negative effect. The same test (i.e., the Kruskal–Wallis test) was used to test the relationship between physical condition and pain based on the SF-MPQ-2. Relative to the variable effect of pain on the patient’s physical state, the Kruskal–Wallis test identified statistically significant differences for all SF-MPQ-2 domains, with a moderate difference observed for continuous and intermittent pain and a large difference observed for the emotional characteristics of the pain as well as for the overall score. The smallest difference (although statistically significant) was identified for neuropathic pain, and this had little impact. It can be noted that pain significantly affected the general physical condition of patients, especially via the emotional components of pain.

Spearman’s Rho non-parametric test was used to determine the extent of the association between a patient’s age and pain assessed using the SF-MPQ-2. At the same time, the effect of pain (i.e., the effect size) was also calculated. On this basis, statistically significant associations were found between intermittent pain, neuropathic pain, and the overall SF-MPQ-2 score; negative correlations were found in all cases. However, for neuropathic pain and the overall score, the correlation was very weak, and the effect was minimal. The strongest correlation was for intermittent pain. It appears that age was only marginally related to SF-MPQ-2 scores. The greatest effect was observed in association with intermittent pain, which occurred with an inverse relationship to age, i.e., the greater the age, the lower the level of this type of pain (Table 5).

## 4. Discussion

To some extent, an unsatisfactory state of pain assessment and management is still present in medical institutions in the Czech Republic. While research has been carried out and there have been publications dealing with this issue, many of the recommendations are yet to be translated into practice [17,18,19]. The importance of transferring current science as it applies to pain management has also been highlighted by the World Health Organization (WHO). In guidelines on pain management, careful initial assessment of patients and correct distinction of acute pain from chronic pain in relation to pharmacological and non-pharmacological treatments are important [20].

Nevertheless, pain remains a significant medical and nursing problem, especially during the postoperative period [21]. Dahlberg, Jaensson, and Nilsson [22] pointed out that most patients in their study who had undergone a one-day surgery contacted the healthcare facility due to pain or to request painkillers. They noted that there was often a need to give or clarify information that the patient should have obtained before surgery or discharge. At the same time, they added that, in half of the cases, the information could have been provided by nurses.

Our results show that, in the surgical departments of the medical institutions in the South Bohemian Region (Czech Republic), patients are evaluated to a slightly higher degree by nurses compared with doctors. Responses also indicate that pain decreases over time after surgery. Intermittent pain remains problematic, and this affected the results obtained with the SF-MPQ-2. Pain characterized as continuous has a strong emotional component as well as a significant negative impact. We also found that pain-related issues tend to decrease with age.

A study by Jang et al. showed [23] that the ability to adequately assess pain in the postoperative period is also associated with personal experiences and professional training. In this sense, we must not forget the role of nursing education, where there is an emphasis on the provision of adequate information to future nurses by educators. The use of standardized tools (e.g., SF-MPQ-2) can help to eliminate bias and improve the accuracy of pain assessment. Jang et al. [23] also reported that nurses put more value on nonverbal pain expression during the postoperative period compared with overt verbal expression. At the same time, however, they try to integrate their clinical experience into the patient’s pain assessment.

## 5. Conclusions

The assessment of patients’ pain is a fundamental skill for nurses working in surgical departments. To some extent, this ability is influenced by clinical experience [23] as well as knowledge acquired during nursing school and continuous education.

The use of standardized tools provides a way to evaluate pain objectively. Our results showed that the SF MPQ-2 can assess the physical and emotional components of pain, both of which are important for overall pain management. On the basis of our results, we suggest that a record sheet that also contains the SF MPQ-2 is used for the assessment of surgical patients’ pain. This sheet was offered to medical institutions where research was done. Now the question is, how do we implement these results into practice to improve the quality of care provided to patients?

## 6. Limitations of the Study

Study participants were selected exclusively from the South Bohemian Region of the Czech Republic. In the case of nurses working in South Bohemian Region, we could not find the number of nurses in each surgical department from the available data. We had to determine the sample size using a statistician based on available data (meaning from the number of surgery department in medical facilities and statutory minimum number of staffing in health services, which is given by Decree of Ministry of Health Czech Republic 99/2012 Sb.). Given the diversity of the Czech population and the differences in healthcare and health facilities, the results may not be entirely generalizable to other areas, countries, or populations. Additionally, we realize that current state of pain can influenced by physical and psychical condition, type of operation or underlying disease as so as pain management. In the present study, a nonparametric bivariate correlation study (Kruskal–Wallis test, Spearman’s Rho non-parametric test) has been conducted. We recognize the risk of bias associated with this type of analysis as we have not controlled for potential confounders.

Nonetheless, the importance of using standardized pain assessment tools in surgical patients and their benefits for the patient are evident.

## Figures and Tables

**Table 1:** Characteristics of the study participants.

**Nurses Working in Surgical Departments**					
Category	Frequency	%	Category	Frequency	%
Age			Gender		
18–29 years	65	25.7	Male	6	2.4
30–39 years	73	28.9	Female	247	97.6
40–49 years	73	28.9			
50 years and over	42	16.6			
**Highest educational attainment**			**Length of experience working in healthcare**		
Secondary	119	47	less than 1 year	13	5.1
Higher Vocational School	51	20.2	1–2 years	23	9.1
University Bachelor’s degree	61	24.1	3–6 years	40	15.8
University Master’s degree	22	8.7	7–15 years	63	24.9
			16 years and over	114	45.1
**Patients hospitalized in surgical departments**					
Category	Frequency	%	Category	Frequency	%
Age			Gender		
15–29 years	27	13.2	Male	91	44.4
30–39 years	54	26.3	Female	114	55.6
40–49 years	45	22			
50–59 years	33	16.1			
60–69 years	23	11.2			
70 years and over	23	11.2			
Number of hospitalizations in the last year			Post-surgical		
1 time	141	68.8	within 24 h	33	16.1
2–3 times	56	27.3	1–2 days	81	39.5
4–5 times	7	3.4	3–5 days	26	12.7
6 times or more	1	0.5	6 days or more	21	10.2
			Pre-surgical	43	21
			Did not answer	1	0.5

**Table 2:** Relationship between the current state of pain and the SF-MPQ-2 assessment (Spearman’s Rho).

		O9	Degree of Variability Explained
**Spearman’s rho**	Continuous Pain	0.274	7.5%
		0.000	
		205	
	Intermittent Pain	0.409	16.7%
		0.000	
		205	
	Neuropathic Pain	0.149	2.2%
		0.033	
		205	
	Affective Dimension	0.198	3.9%
		0.004	
		205	
	SF-MPQ-2	0.425	18.1%
		0.000	
		205	

**Table 3:** Evaluation of the relationship between the current pain state and selected variables.

**Monitored Relationship**						
**Duration of Pain at a Given Intensity**						
**Intensity of Pain**	**Time of Pain**					
	**Less than 24 h**	**2–3 days**	**4–6 days**	**7 days–1 Month**	**more than 1 Month**	**Sum**
0–3	35	8	3	2	3	51
no pain, weak pain	−68.60%	−15.70%	−5.90%	−3.90%	−5.90%	−100%
4–6	57	48	15	2	6	128
moderate pain	−44.50%	−37.50%	−11.70%	−1.60%	−4.70%	−100%
7–10	9	10	4	3	0	26
severe pain, highest possible level of pain	−34.60%	−38.50%	−15.40%	−11.50%	0%	−100%
**Impact of Current Pain Status on the patient’s Mental State**						
**Intensity of Pain**	**Impact on Mental State**					
	**Maximum**	**High**	**Medium**	**Low**	**None**	**Sum**
0–3	1	5	7	11	27	51
no pain, weak pain	−2.00%	−9.80%	−13.70%	−21.60%	−52.90%	−100%
4–6	7	25	23	26	47	128
moderate pain	−5.50%	−19.50%	−18.00%	−20.30%	−36.70%	−100%
7–10	4	9	7	4	2	26
severe pain, greatest possible level of pain	−15.40%	−34.60%	−26.90%	−15.40%	−7.70%	−100%
**Impact of Current Pain Status on the Patient’s Physical Condition**						
**Intensity of Pain**	**Physical Impact Rate**					
	**Maximum**	**Much**	**Medium**	**Low**	**None**	**Sum**
0–3	2	6	4	17	22	51
no pain, weak pain	−3.90%	−11.80%	−7.80%	−33.30%	−43.10%	−100%
4–6	12	22	35	43	16	128
moderate pain	−9.40%	−17.20%	−27.30%	−33.60%	−12.50%	−100%
7–10	6	8	9	2	1	26
severe pain, greatest possible level of pain	−23.10%	−30.80%	−34.60%	−7.70%	−3.80%	−100%

**Table 4:** Relationship between physical and mental states and the level of pain evaluated using the SF-MPQ-2.

**Association between Mental State and Pain Based on the SF-MPQ-2**					
	**Continuous Pain**	**Intermittent Pain**	**Neuropathic Pain**	**Affective Dimension**	**SF-MPQ-2**
Chi-Square	17.562	8.081	6.427	38.073	30.574
Df	4	4	4	4	4
Asymp. Sig	0.002	0.089	0.169	0.000	0.000
Effect size ŋ^2^	00.09			00.19	00.15
Evaluation of the effect	Medium			Large	Large
**Association between Physical Condition and Pain Based on the SF-MPQ-2**					
	**Continuous Pain**	**Intermittent Pain**	**Neuropathic Pain**	**Affective Dimension**	**SF-MPQ-2**
Chi-Square	22,283	22,742	12,708	40,972	48,532
Df	4	4	4	4	4
Asymp. Sig	0.000	0.000	0.013	0.000	0.000
Effect size ŋ^2^	00.11	00.11	00.06	00.20	00.24
Evaluation of the effect	Medium	Medium	Small	Large	Large

*Df*—degrees of freedom.

**Table 5:** Association of the total SF-MPQ-2 score with patient age.

			Age	Degree of Variability Explained
**Spearman’s rho**	Continuous Pain	Correlation Coefficient	−0.062	0.4%
		Sig. (2-tailed)	0.376	
		N	205	
	Intermittent Pain	Correlation Coefficient	−0.237	5.6%, i.e., small effect
		Sig. (2-tailed)	0.001	
		N	205	
	Neuropathic Pain	Correlation Coefficient	−0.159	2.5%, i.e., very small effect
		Sig. (2-tailed)	0.023	
		N	205	
	Affective Dimension	Correlation Coefficient	0.069	0.5%
		Sig. (2-tailed)	0.327	
		N	205	
	SF-MPQ-2	Correlation Coefficient	−0.141	2.0%, i.e., very small effect
		Sig. (2-tailed)	0.044	
		N	205	

## Data Availability

The data are not publicly available. Outputs from the grant project GAJU 058/2018/S are available in the database Research, development and innovation information system. Available on: https://www.isvavai.cz/ (accessed on 4 September 2020).
